# Lupus Never Fails to Deceive US: A Case of Rowell's Syndrome

**DOI:** 10.1155/2020/8884230

**Published:** 2020-09-22

**Authors:** Ana B. Arevalo, Rawann Nassar, Satyam Krishan, Priyanka Lakshmanan, Maria Salgado, Priya Chokshi

**Affiliations:** ^1^Department of Medicine, Mount Sinai Morningside and Mount Sinai West, Icahn School of Medicine at Mount Sinai, New York, NY, USA; ^2^Punjab Institute of Medical Sciences, Jalandhar, Punjab, India; ^3^Department of Medicine, Jacobi Medical Center, Albert Einstein College of Medicine, Bronx, NY, USA; ^4^Department of Rheumatology, Mount Sinai Morningside and Mount Sinai West, Icahn School of Medicine at Mount Sinai, New York, NY, USA

## Abstract

**Background:**

Rowell's syndrome is comprised of the presentation of erythema multiforme- (EM-) like lesions in association with lupus erythematosus (LE), along with serologies of speckled antinuclear antibodies (ANAs), positive rheumatoid factor (RF), positive anti-La/anti-Ro, and the clinical finding of chilblains. As per the redefined criteria by Zeitouni et al., three major criteria in addition to at least 1 minor criterion are necessary for diagnosis. *Case Presentation*. A 20-year-old male presented with a one-week history of worsening nonpruritic erythematous maculopapular skin rash (resembling EM) which appeared on the face and subsequently spread to the trunk, arms, legs, palms, and soles. There was no mucosal involvement. At the onset of rash, the patient reported headaches, associated with photosensitivity and intermittent fevers. Workup for viral meningitis yielded negative results. Laboratory investigation revealed mild anemia, elevated inflammatory markers, a positive ANA with speckled pattern, a positive anti-Ro/SSA, anti-La/SSB antibodies, and a positive rheumatoid factor (RF). Lupus anticoagulant antibody was positive along with a low-positive anticardiolipin IgM antibody and a negative beta-2 glycoprotein antibody. Anti-dsDNA, anti-Smith, anti-Jo-1, anti-centromere, and anti-Scl-70 antibodies were negative. Hepatitis serologies, herpes simplex virus 1 and 2, mycoplasma, Epstein–Barr virus, HIV, and parvovirus B19 were negative. Left arm skin biopsy demonstrated vacuolar interface dermatitis and positive colloidal iron stain suggestive of dermal mucin deposition, favoring the diagnosis of cutaneous collagen vascular disease. Cutaneous lesions improved with administration of oral prednisolone.

**Conclusion:**

Rowell's syndrome should be considered in patients who present with cutaneous LE and lesions resembling EM. Further serological markers should be pursued in the absence of obvious EM-precipitating factors.

## 1. Introduction

Rowell's syndrome describes the constellation of erythema multiforme- (EM-) like lesions in association with lupus erythematosus (LE), along with the findings of speckled ANA, positive rheumatoid factor (RF), positive anti-La/anti-Ro, and the clinical finding of chilblains.

The coexistence of EM-like lesions and LE was first described by Scholtz in 1922; however, the distinct entity of Rowell's syndrome was then described in 1963 by Rowell et al. who recognized particular serologic markers of patients with this overlap [1].

Ever since its proposal in 1963, there have been numerous reports of cases relating their findings to Rowell's syndrome, many of which were later found to lack the criteria originally described by Rowell. This led to a revision of diagnostic criteria, which were redefined by Zeitouni et al. in the year 2000 to account for the inconsistency of certain criteria as compared to others. As redefined, major criteria include the coexistence of lupus erythematosus (SLE or discoid lupus erythematosus) or subacute cutaneous lupus erythematosus, with EM-like lesions with or without mucosal involvement and finally speckled pattern of ANA. Minor criteria include clinical finding of chilblains, positive anti-Ro or anti-La antibodies, and lastly positive rheumatoid factor (RF). In order to make the diagnosis, all three major criteria, as well as at least 1 minor criterion, must be met [2, 3].

Whether Rowell's syndrome exists has remained a matter of debate among physicians with some believing it to be a mere coincidental occurrence of EM in patients with LE while some believing it to be a cutaneous manifestation of lupus.

We report the case of a healthy 20-year-old male who presented with EM-like lesions, positive RF, positive ANA with a speckled pattern, and positive anti-Ro antibody titer consistent with diagnosis of Rowell's syndrome.

## 2. Clinical Presentation

**Figure 1:**
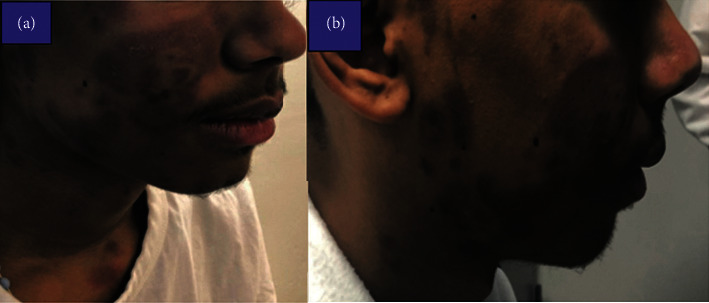
Well-defined, nonblanching confluent papules and plaques in the face.

**Figure 2:**
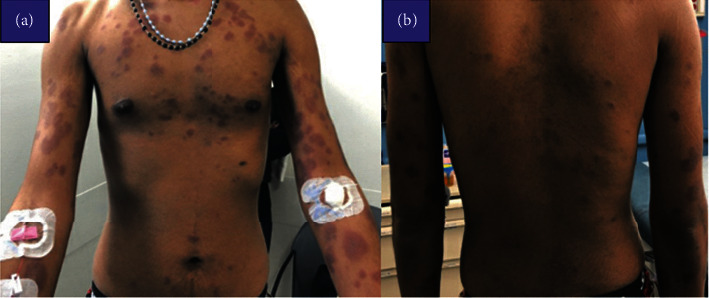
Well-defined, nonblanching confluent papules and plaques in the trunk.

**Figure 3:**
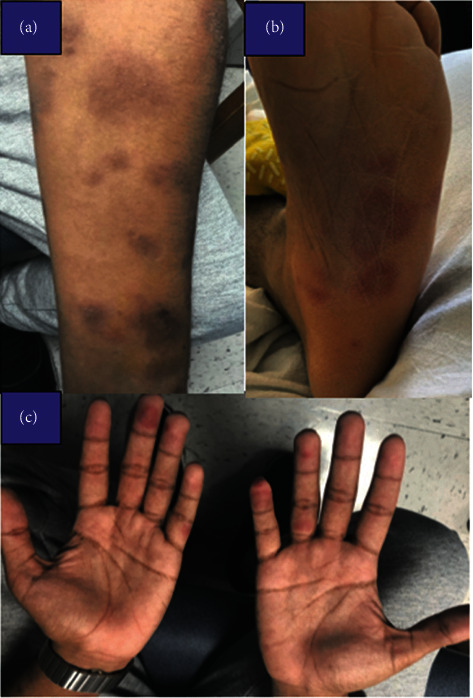
Well-defined, nonblanching confluent papules and plaques in the arm (a), soles (b), and palms (c).

A 20-year-old male with no prior medical diagnoses presented with a one-week history of worsening nonpruritic erythematous maculopapular skin rash which initially appeared on his face (Figure 1) and subsequently spread to his trunk (Figure 2) followed by his arms, legs, palms, and soles (Figure 3). At the onset of the rash, the patient reported associated headaches, photosensitivity, as well as intermittent fevers. Workup for viral meningitis yielded negative results, and the clinical picture was most consistent with a viral exanthem. He recovered spontaneously with slight resolution of the rash and, as a result, refused skin biopsy.

One month later, he presented once again with worsening of the same nonpruritic rash, associated with neck pain, photophobia, headache, low-back pain, fatigue, anorexia, as well as intermittent fevers with a peak temperature of 102F. Examination revealed generalized well-defined nonpruritic, nonblanching confluent targetoid papules and plaques (resembling EM) in various stages of healing. The lesions were located primarily on the torso, back, arms, legs, palms, soles, and lips. Newer lesions appeared erythematous, while older lesions appeared darker. The lesions located on the forearms and face were noted to exhibit small areas of fine scaling. There was no evidence of mucosal involvement.

In addition to the initial workup for infectious etiology and viral as well as bacterial meningitis (performed since the initial presentation was fever, photosensitivity, and headache), laboratory investigation revealed mild anemia (Hb 12.6 g/dL), elevated erythrocyte sedimentation rate (ESR) of 75 mm/h, C-reactive protein (CRP) of 11.05 mg/L, a positive ANA titer of >1 : 1280 with speckled pattern, a positive anti-Ro/SSA, anti-La/SSB titers of >8.0, and a positive RF of 34 IU/ml. Lupus anticoagulant antibody was positive (>44.4 sec), along with a low-positive anticardiolipin IgM antibody (>13 U/ml) and a negative beta-2 glycoprotein antibody. Anti-dsDNA, anti-Smith, anti-Jo-1, anti-centromere, and anti-Scl-70 antibodies were negative. Serum chemistry and urinalysis were within normal limits. Hepatitis B virus (HBV), hepatitis C virus (HCV), herpes simplex virus (HSV) 1 and 2, mycoplasma, Epstein–Barr virus (EBV), human immunodeficiency virus (HIV), and parvovirus B19 were negative.

The patient then underwent a left arm skin biopsy, which demonstrated vacuolar interface dermatitis and positive colloidal iron stain suggestive of dermal mucin deposition, favoring the diagnosis of cutaneous collagen vascular disease.

Cutaneous lesions improved with administration of oral prednisolone.

## 3. Discussion

This case highlights the importance of maintaining a diagnostic suspicion for Rowell's syndrome in LE patients who present with EM-like lesions. Lupus anticoagulant antibody was positive in our patient, but he did not meet criteria for antiphospholipid syndrome (APS). It should be noted, however, that a rare association between APS and Rowell's syndrome has been described. RS in association with LE and APS could be a distinct entity, or RS could be a spectrum of clinical and immunologic phenomenon of which APS may be one. Further characterization requires analysis of coagulation parameters in patients [4].

The important learning points from this case are twofold. First and foremost, although Rowell's syndrome is a recognized syndrome in patients with a diagnosis of LE who present with a certain correlation of clinical symptoms and findings, a degree of suspicion should be maintained in those who do not have a prior diagnosis of LE, such as in our patient.

Patients with cutaneous LE may develop coincidental EM. However, if characteristic serological abnormalities are present and there is no obvious precipitating event, the association is known as Rowell's syndrome.

Since the first report of RS, not more than 71 cases have been reported in the English literature; nonetheless, a recent review demonstrated that most of the reported cases did not fulfill all the diagnostic criteria of Rowell's original description, especially the presence of RF and anti-La antibody [5].

The current diagnostic criteria help us distinguish RS from other causes of EM which include drugs and infections. Specifically, in patients with LE, possible causes for EM in the presence of an inciting event could be NSAIDs if used for management of arthritis. Use of immunosuppressants can predispose patients to infections (tuberculosis, herpes-simplex virus, cytomegalovirus, and fungal infections) which are independent triggers for EM [6].

Other possible differentials for EM include urticaria, Stevens–Johnson syndrome (SJS), fixed drug eruption, and cutaneous small vasculitis [7]. These conditions can be differentiated using a combination of clinical, histologic, and laboratory findings.

Our patient met 3 major and 2 minor criteria thus establishing a diagnosis of RS (Table 1). A detailed history and thorough physical examination with particular attention to the extremities in search for evidence of chilblains can help narrow the differential and establish the diagnosis of RS. Histology can further aid in diagnosis of RS. Some findings common to the reported cases of RS include keratinocyte necrosis, perivascular dermal lymphocytic infiltrate, and vacuolar degeneration of the dermal-epidermal junction [8–11]. The left arm skin biopsy in our patient showed vacuolar interface dermatitis and dermal mucin deposition favoring the diagnosis of cutaneous collagen vascular disease.

Diagnosis of this condition is prudent for further management and appropriate monitoring of possible complications of LE. Most reports show a successful treatment using systemic steroids and additional immunosuppressive drugs such as azathioprine and cyclosporine. Some studies also reported the use of topical steroids in the management of RS with variable response [8, 9]. Zeitouni et al. also described a case of RS that responded to treatment with dapsone [3]. In the case of our patient, the cutaneous lesions improved with administration of oral prednisolone.

## 4. Conclusions

Rowell's syndrome should be considered in patients who present with cutaneous LE and lesions resembling EM. Further serological markers should be pursued in the absence of obvious EM-precipitating factors. This presentation is unique since Rowell's syndrome has been described rarely in men; most of the cases reported in the literature are predominantly middle-aged women.

## Figures and Tables

**Table 1:** Criteria for diagnosis of Rowell syndrome [3].

Major criteria	Minor criteria
(i) Lupus erythematosus (LE): systemic LE, discoid LE, or subacute cutaneous LE	(i) Chilblains
(ii) Erythema multiforme-like lesions (with/without involvement of the mucous membranes)	(ii) Anti-Ro or anti-La antibody
(iii) Speckled pattern of ANA	(iii) Positive rheumatoid factor

## Data Availability

The data used to support the findings of this study are available from the corresponding author on request.
